# Lysophosphatidylcholine Impairs the Mitochondria Homeostasis Leading to Trophoblast Dysfunction in Gestational Diabetes Mellitus

**DOI:** 10.3390/antiox13081007

**Published:** 2024-08-19

**Authors:** Shao-Chi Hung, Te-Fu Chan, Hsiu-Chuan Chan, Chia-Ying Wu, Mei-Lin Chan, Jie-Yang Jhuang, Ji-Qin Tan, Jia-Bin Mei, Shi-Hui Law, Vinoth Kumar Ponnusamy, Hua-Chen Chan, Liang-Yin Ke

**Affiliations:** 1 Department of Medical Laboratory Science and Biotechnology, College of Health Sciences, Kaohsiung Medical University, Kaohsiung 807378, Taiwan; wisley819@gmail.com (S.-C.H.); tanjiqin1115@gmail.com (J.-Q.T.); dcdc96028@gmail.com (J.-B.M.); shlaw_0909@hotmail.com (S.-H.L.); 2 Graduate Institute of Medicine, College of Medicine & Drug Development and Value Creation Research Center, Kaohsiung Medical University, Kaohsiung 807378, Taiwan; tfchan@kmu.edu.tw; 3 Department of Obstetrics and Gynecology, Kaohsiung Medical University Hospital, Kaohsiung 807377, Taiwan; 4 PhD Program in Life Science, College of Life Science, Kaohsiung Medical University, Kaohsiung 807378, Taiwan; earth1981709@hotmail.com (H.-C.C.); kumar@kmu.edu.tw (V.K.P.); 5 The Master Program of AI Application in Health Industry, College of Health Sciences, Kaohsiung Medical University, Kaohsiung 807378, Taiwan; xxx1020828@gmail.com; 6 Division of Thoracic Surgery, Department of Surgery, MacKay Memorial Hospital, MacKay Medical College, Taipei 104217, Taiwan; t102@mmc.edu.tw; 7 Department of Medicine, MacKay Medical College, New Taipei 252005, Taiwan; superelud@gmail.com; 8 Department of Pathology, Mackay Memorial Hospital, Tamsui Branch, New Taipei 251404, Taiwan; 9 Department of Medicinal and Applied Chemistry & Research Center for Precision Environmental Medicine, Kaohsiung Medical University, Kaohsiung 80708, Taiwan; 10 Department of Medical Laboratory Science, College of Medicine, I-Shou University, Kaohsiung 824005, Taiwan; 11 Center for Lipid Biosciences, Department of Medical Research, Kaohsiung Medical University Hospital, Kaohsiung 807377, Taiwan; 12 Department of Laboratory Medicine, Kaohsiung Medical University Hospital, Kaohsiung 807377, Taiwan

**Keywords:** lysophosphatidylcholine (LPC), gestational diabetes mellitus (GDM), mitochondrial dysfunction, hypoxia-induced factor-1 alpha (HIF-1α), placenta, trophoblast

## Abstract

Gestational diabetes mellitus (GDM) is a common pregnancy disorder associated with an increased risk of pre-eclampsia and macrosomia. Recent research has shown that the buildup of excess lipids within the placental trophoblast impairs mitochondrial function. However, the exact lipids that impact the placental trophoblast and the underlying mechanism remain unclear. GDM cases and healthy controls were recruited at Kaohsiung Medical University Hospital. The placenta and cord blood were taken during birth. Confocal and electron microscopy were utilized to examine the morphology of the placenta and mitochondria. We determined the lipid composition using liquid chromatography-mass spectrometry in data-independent analysis mode (LC/MS^E^). In vitro studies were carried out on choriocarcinoma cells (JEG3) to investigate the mechanism of trophoblast mitochondrial dysfunction. Results showed that the GDM placenta was distinguished by increased syncytial knots, chorangiosis, lectin-like oxidized low-density lipoprotein (LDL) receptor-1 (LOX-1) overexpression, and mitochondrial dysfunction. Lysophosphatidylcholine (LPC) 16:0 was significantly elevated in the cord blood LDL of GDM patients. In vitro, we demonstrated that LPC dose-dependently disrupts mitochondrial function by increasing reactive oxygen species (ROS) levels and HIF-1α signaling. In conclusion, highly elevated LPC in cord blood plays a pivotal role in GDM, contributing to trophoblast impairment and pregnancy complications.

## 1. Introduction

Gestational diabetes mellitus (GDM) is dysglycemia that develops or is first detected during pregnancy [1], with a fasting glucose level greater than 5.1 mmol/L (92 mg/dL) or abnormal 75 g oral glucose tolerance test (OGTT: one-hour > 10 mmol/L (180 mg/dL) or two-hour > 8.5 mmol/L (153 mg/dL)) performed between 24 and 28 weeks of pregnancy [2]. GDM may lead to unfavorable pregnancy outcomes such as pre-eclampsia, cesarean delivery, metabolic syndrome, and atherosclerosis [3,4]. Besides, GDM fetuses are more likely to develop macrosomia, which increases the risk of fatality [5,6]. Several cohort studies have indicated that GDM prevalence has increased globally in recent years [7,8,9,10], and it has become one of the major concerns in women’s health [11].

During early pregnancy, the implantation site suffers transient hypoxia, which allows extravillous trophoblasts to invade the uterine decidua and myometrium [12,13]. Even so, persistent placental hypoxia is a substantial contributor to pregnancy complications [14,15]. Up-regulation of placental hypoxia-inducible factor 1-alpha (HIF-1α) is a determining pathogenic mechanism promoting disease progression [16]. Furthermore, the soluble form of lectin-like oxidized LDL (oxLDL) receptor-1 (LOX-1) represents a novel biomarker of neonatal hypoxic-ischemic encephalopathy [17]. Highly expressed LOX-1 in the placenta and endothelium is associated with oxLDL uptake [18,19] and mitochondrial dysfunction [20,21]. Ultimately, impaired mitochondria cause oxidative stress and homeostasis disruption, which results in the manifestation of placenta accreta spectrum (PAS) disorder [22].

The placenta is a multifaceted transient organ that connects mothers and growing fetuses [23,24]. It supplies the fetus with oxygen, lipoproteins, and nutrients due to its enormous surface area, allowing energy and metabolite exchanges [25]. The trophoblast is the predominant cell type in the placenta, forming the outer layers and supplying nutrition to the embryo [26]. Hyperglycemia impairs placental mitochondrial function while altering lipid metabolism and hormone secretion [27]. Conversely, the alteration of lipid metabolism that contributes to pathogenesis has yet to be defined. GDM has also been linked to structural alterations in the human placenta, including increased intervillous space and terminal villi, syncytiotrophoblast number fibrinoid areas, and chorangiosis [28]. These alterations may cause functional changes in this organ, reducing the developing fetus’s well-being [29].

GDM is associated with metabolic alterations of lipid profile [30]. Notably, the documented elevation in triglycerides (TG) occurs in the first trimester and persists into the third, significantly earlier than hyperglycemia [31,32]. On the contrary, the levels of total cholesterol (TC), high-density lipoprotein cholesterol (HDL-C), and low-density lipoprotein cholesterol (LDL-C) are reduced in GDM mothers [31]. Lipidomic studies have revealed that a broad spectrum of lipid species is altered in GDM plasma, including TG, diglycerides, sphingolipids, and lysophosphatidylcholines (LPC) [33,34,35,36]. The majority of GDM plasma lipidomic profiles are similar to those of type 2 diabetes mellitus (T2D), contributing to the transition from GDM to T2D [35]. Nevertheless, the lipidome in cord blood and placenta and the pathophysiology of lipids in GDM remain undefined.

In order to better understand the pathophysiology in the placenta, this study aimed to identify lipid components in the cord blood of GDM. Moreover, we identified the etiological mechanism that disrupts mitochondria homeostasis, resulting in trophoblast dysfunction in GDM. In doing so, it could enable us to discover novel therapeutic approaches for GDM.

## 2. Materials and Methods

### 2.1. Study Design and Sample Collection

The study procedures were approved by the institutional review board (IRB) of the Kaohsiung Medical University Hospital (KMUH), Kaohsiung, Taiwan (KMUHIRB-SV(II)-20170081). Participants were recruited on the first visit to KMUH from March 2018 to December 2022 and followed up throughout their pregnancy. The inclusion criteria were pregnant women and those over 20 years old. In contrast, the exclusion criteria were patients with cardiovascular disease, metabolic disorders, and diabetes. Participants received consecutive blood testing for biochemical profiles in each trimester. In the second trimester (24–28 weeks), the participants received a 75 g OGTT was used to diagnose GDM and non-gestational diabetes (NGDM) using the IADPSG criteria (fasting glucose > 92 mg/dL, 1 h postprandial glucose > 180 mg/dL, 2 h postprandial glucose > 153 mg/dL). In the present study, we registered detailed information from 12 Taiwanese women with a signed consent document (*n* = 6 for the GDM group and *n* = 6 for NGDM) (Table 1). Blood samples from the umbilical cord were taken during the delivery operation. Placental tissues were collected within one hour of delivery and preserved in 4% paraformaldehyde (Thermo Fisher Scientific; Waltham, MA, USA).

### 2.2. Histology Analysis

The preserved tissue samples were dehydrated, cleared with xylene, and embedded in paraffin. Before staining, the paraffin block was sectioned and deparaffinized. Hematoxylin and eosin (H&E) staining was performed according to the manufacturer’s instructions (BioTnA; Kaohsiung, Taiwan). The images were taken using a Nikon Ci-L Plus microscope (Nikon; Tokyo, Japan), and later images were analyzed at the pathology department of MacKay Memorial Hospital for syncytial knots, chorionic stroma rich in secondary cells, stem villi with indented edge, extravillous fibrinoid between terminal villi, and so on.

For immunohistochemistry staining, the placental tissue slides were retrieved in 10 mM sodium citrate (BioTnA; Kaohsiung, Taiwan) and stained with primary antibody against LOX-1 (Thermo Fisher Scientific; Waltham, MA, USA) for 2 h at room temperature. A horseradish peroxidase (HRP)-conjugated anti-rabbit probe was used and detected with 3,3′-diaminobenzidine (DAB) (BioTnA; Kaohsiung, Taiwan). After counterstaining the nucleus with hematoxylin, images were taken with a Nikon Ci-L Plus microscope.

For immunofluorescence staining, the slides were retrieved in 10 mM sodium citrate and stained with primary antibodies against the mechanochemical GTPase optic atrophy 1 (OPA1, Cell Signaling Technology; Danvers, MA, USA) and mitochondrial import receptor subunit (TOM20) homolog (Abcam; Cambridge, UK). Anti-mouse 488 and anti-rabbit 594 (BioTnA; Kaohsiung, Taiwan) were performed as secondary antibodies, respectively. The nucleus was counterstained with DAPI 4′,6-diamidino-2-phenylindole (Thermo Fisher Scientific; Waltham, MA, USA) and photographed using a Zeiss LSM700 confocal microscope (Zeiss; Oberkochen, Germany).

### 2.3. Transmission Electron Microscopy (TEM)

Fresh placenta samples were maintained in a 0.1M phosphate-buffered saline (PBS) solution (pH 7.4) containing 2.5% glutaraldehyde and 2% paraformaldehyde. After the wash, samples were secondary fixed using 2% osmium tetroxide (OsO4) and stained with a 50% uranyl acetate solution in ethanol. Stained samples were examined with an HT7700 transmission electron microscope (Hitachi; Tokyo, Japan) at 100 kV accelerating voltage and 0.204 nm resolution. Digital images were captured with an 8-million-pixel camera system.

### 2.4. Human LDL Isolation

Cord blood samples from 12 Taiwanese women were taken during labor in anticoagulant tubes containing ethylenediaminetetraacetic acid (EDTA) (BD; Franklin Lakes, NJ, USA). Platelet-free plasma samples were collected using two centrifugations: 3000× *g* rpm for 10 min and 800× *g* for 20 min. Each chylomicron subfraction was removed from the platelet-free plasma by overlaying it with pure water and ultracentrifuging at 45,000× *g* rpm for 2 h at 4 °C. Each low-density lipoprotein (Density = 1.019–1.063 g/mL) subfraction was isolated using sequential potassium bromide (Merck; Darmstadt, Germany) density solution at 65,000 rpm for 24 h with a Type 90 Ti Fixed-Angle Titanium Rotor (Beckman; Brea, CA, USA). The extracted LDL samples were dialyzed with 0.02 M Tris buffer containing 0.5 mM EDTA and stored at 4 °C.

### 2.5. Lipid Extraction

The 100 µg dialyzed LDL samples from 12 Taiwanese women were further dissolved into 800 µL of MS-grade water (PURELAB Option-Q; Oxford, UK) for lipid extraction. The approach was conducted with a biphasic combination of chloroform (J.T. Baker, Avantor; Radnor, PA, USA) and methanol (Thermo Fisher Scientific; Waltham, MA, USA). Methanol and chloroform were added to achieve the final MeOH:CHCl_3_:H_2_O ratio of 2:2:1.8. After centrifugation at 3000× *g* rpm for 10 min at 4 °C, the chloroform layer was aspirated from the bottom of the tube using a 10 cm needle and collected in a glass vial. Each of the lipid extracts was evaporated with air nitrogen and resuspended with 200 µL lipids solution comprising isopropanol (Merck; Darmstadt, Germany), acetonitrile (J.T. Baker, Avantor; Radnor, PA, USA), and MS-grade water (PURELAB Option-Q; Wembley, UK) in the ratio of 2:1:1.

### 2.6. LC/MS^E^ Analysis

Total lipid profiling of each LDL-lipid extract from 12 Taiwanese women was carried out using ultra-pure liquid chromatography (ACQUITY^®^ UPLC) and XEVO G2 QTOF mass spectrometry (Waters Corporation; Milford, MA, USA). A charged surface hybrid C18 column (CSH, 2.1 mm × 10 cm, 1.7 µm, Waters Corporation; Milford, MA, USA) separated molecules based on polarity. The UPLC separation was programmed at a flow rate of 0.1 mL/min for 20 min at 55 °C. The mobile phase A was created by mixing acetonitrile (ACN, Merck; Darmstadt, Germany) with MS-grade water at a 6:4 ratio and additionally contained 10 mM ammonium formate and 0.1% formic acid (Merck; Darmstadt, Germany). The mobile phase B was a solution containing 10 mM ammonium formate and 0.1% formic acid in isopropanol (IPA, Merck; Darmstadt, Germany) and ACN at a 90:10 ratio. The electrospray ionization interface (ESI) technology was utilized to ionize lipid molecules. The signals of ionized fragmentations were obtained by switching the voltage between low (4 eV) and high (35–55 eV) energies using leucine enkephalin for real-time calibration. Data were analyzed by Progenesis QI^®^ (Waters Corporation; Milford, MA, USA) and visualized by MetaboAnalyst 6.0.

### 2.7. Cell Culture

Human placental JEG3 cells were purchased from the Bioresource Collection and Research Center (BCRC; Hsinchu, Taiwan). Cells were maintained in minimum essential media (MEM) with 2 mM L-glutamine and 1 mM sodium pyruvate (Gibco, Thermo Fisher Scientific; Waltham, MA, USA) and 10% fetal bovine serum (Invitrogen, Thermo Fisher Scientific; Waltham, MA, USA) in an incubator at a constant temperature of 37 °C and 5% CO_2_.

The cells were treated subsequentially with 16:0 LPC (Avanti Polar Lipids; Alabaster, AL, USA). Before treatment, the LPC solvent was dried by a nitrogen gas evaporator (Eyela MG-2200; Tokyo, Japan). After resuspending LPC with the MEM medium containing 2% FBS and 4.5 g/L glucose, cells were incubated with LPC for 6 or 24 h. CAY10585 (Cayman Chemical; Ann Arbor, MI, USA), the HIF-1α inhibitor, was pre-treated to the cell for 2 h before incubating with LPC for 24 h.

### 2.8. Confocal Microscopy

JEG3 cells were seeded on a sterile coverslip (Paul Marienfeld GmbH & Co., Laida-Königshofen, Germany) at a density of 1.2 × 10^4^/cm^2^ for 24 h. Following LPC treatment, cells were labeled with 100 nM MitoTracker™ Red CMXRos, 100 nM MitoTracker™ Green FM, and 1 mM MitoSOX™ Red (Thermo Fisher Scientific; Waltham, MA, USA) in the media. After a PBS wash, the cells were fixed with 4% paraformaldehyde (Thermo Fisher Scientific; Waltham, MA, USA) for 20 min at 4 °C. The coverslip was mounted on a slide using ProLong™ Gold Antifade Mountant (Thermo Fisher Scientific; Waltham, MA, USA) and stored at 4 °C. The high-resolution images were acquired with a Zeiss LSM700 confocal microscope (Zeiss; Oberkochen, Germany).

### 2.9. Mitochondria Isolation

After LPC treatment, JEG3 cells were collected at 1.5 × 10^6^ cells for further processing. The mitochondria were isolated using a cell fractionation kit (Abcam; Cambridge, UK). To evaluate isolation efficiency, Western blotting was performed against specific targets, α-tubulin (Proteintech; Rosemont, IL, USA) and voltage-dependent anion channels (VDAC; Cell Signaling Technology; Danvers, MA, USA).

### 2.10. Western Blot Analysis

JEG3 cells were treated with either sequential concentrations of LPC or control. Cells were harvested with radioimmunoprecipitation assay buffer (RIPA) lysis buffer containing 150 mM sodium chloride (Honeywell International Inc.; Charlotte; NC, USA), 50 mM Tris-base (J.T. Baker, Avantor; Radnor, PA, USA), 0.1% sodium dodecyl sulfate (SDS; VWR, Avantor; Radnor, PA, USA), 1% NP-40 (Thermo Fisher Scientific; Waltham, MA, USA), and 0.5% sodium deoxycholate (Sigma Aldrich, Merck; Darmstadt, Germany) with cOmplete™ Protease Inhibitor Cocktail (Roche; Basel, Switzerland). The concentration of protein lysate was measured using Pierce™ BCA Protein Assay Kits (Thermo Fisher Scientific; Waltham, MA, USA). The polyacrylamide separating gel was composed of 1.5 M TRIS Hydrochloride (Tris-HCl), pH 8.8 (Biomate^TM^, Rainbow Biotechnology; Taipei, Taiwan), 30% Acrylamide/Bis Solution (Bio-Rad Laboratories; Hercules, CA, USA), pure water, ammonium persulfate, 10% sodium dodecyl sulfate (SDS) and N,N,N′,N′-Tetramethyl ethylenediamine (TEMED; Sigma Aldrich, Merck; Darmstadt, Germany). The stacking gel was substituted with 0.5 M Tris-HCl pH 6.8 (Biomate^TM^, Rainbow Biotechnology; Taipei, Taiwan).

Following the mix with 5× sample dye, the samples were incubated at 95 °C for 5 min, and electrophoresis with the buffer consisting of 25 mM Tris-base, 192 mM glycine (J.T. *Baker*, *Avantor*; Radnor, PA, USA), and 10% SDS. The protein on the gel was transferred onto the polyvinylidene difluoride membrane (PVDF; Merck; Darmstadt, Germany) and blocked with the 5% milk in Tris-buffered Saline containing 0.1% Tween 20 (TBST; Sigma Aldrich, Merck; Darmstadt, Germany). The membrane was incubated with primary antibody for 1 h: HIF-1*α*, beta-actin (GeneTex; Irvine, CA, USA), α-tubulin (Proteintech; Rosemont, IL, USA), OPA1, VDAC (Cell Signaling Technology; Danvers, MA, USA), LOX-1 (Thermo Fisher Scientific; Waltham, MA, USA). After TBST wash, the HRP-conjugated anti-rabbit *immunoglobulin* G (IgG) and HRP-conjugated anti-mouse IgG (Sigma Aldrich, Merck; Darmstadt, Germany) were carried out as secondary antibodies for one hour of shaking incubation. The signal was acquired using a LumiLong Plus Chemiluminescence detection kit (T-pro Biotechnology; New Taipei, Taiwan) and visualized using the ChemiDoc Image System (Bio-Rad *Laboratories*; Hercules, CA, USA).

### 2.11. Statistic Analysis

Continuous responses are displayed as the mean ± standard error of the mean (SEM), whereas discrete responses are provided as relative frequency. All data statistics and figures were analyzed and generated using GraphPad Prism Software version 9 (GraphPad Software Inc.; La Jolla, CA, USA). Student’s *t*-test and Mann–Whitney U test were used to assess group differences. Values were considered statistically significant at *p* < 0.05. Statistical significance is indicated by the following symbols: * *p* < 0.05, ** *p* < 0.01, *** *p* < 0.001; for inhibitor effect, # *p* < 0.05, ## *p* < 0.01, ### *p* < 0.001. The principal component analysis (PCA) for component importance statistics was operated using Python version 3.12.4.

## 3. Results

### 3.1. Modification of GDM Placenta with Abnormal Structure and Increased LOX-1 Expression

**Figure 1:**
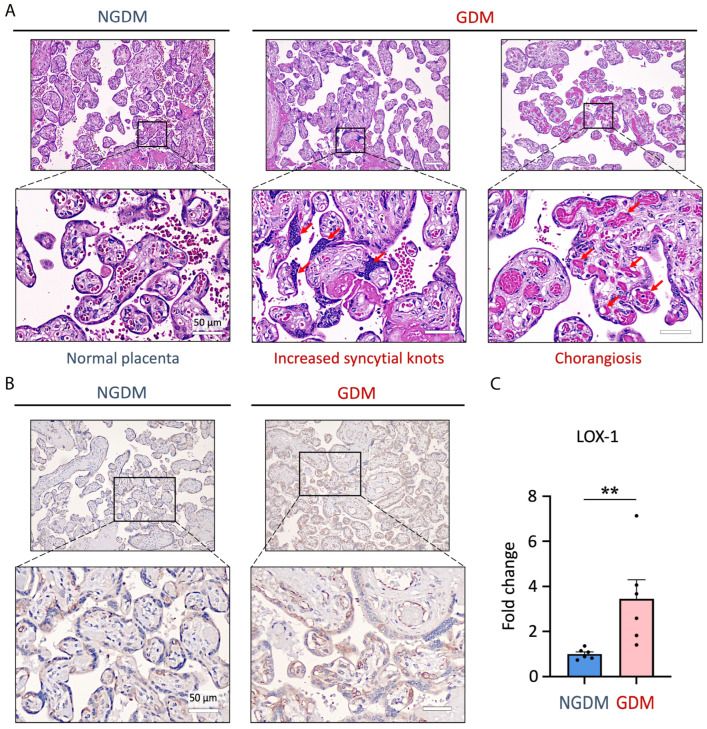
The GDM placenta had aberrant structures and LOX-1 overexpression. (**A**) H&E staining of the placenta in GDM revealed more syncytial knots and chorangiosis than in healthy controls (*n* = 6, six placentas from six different donors for each group). Scale bar represents 50 µm. The red arrow represents placental lesion. (**B**) Representative immunohistochemical staining for LOX-1 expression in healthy and GDM patients (*n* = 6). Scale bar: 50 µm. (**C**) LOX-1 expression increased 3.4-fold in GDM compared to healthy controls (*n* = 6). ** *p* < 0.01. Abbreviations: GDM: gestational diabetes mellitus; NGDM: non-gestational diabetes healthy controls; LOX-1: lectin-like oxidized LDL receptor-1.

Previous studies have shown that LOX-1 overexpression in preeclampsia-syncytiotrophoblast-derived extracellular vesicles promotes endothelial dysfunction, indicating that the LOX-1 pathway may be involved in pregnancy complications [29,37]. To determine if LOX-1 also plays a role in GDM, we examined LOX-1 expression and its association with placental insufficiency. Figure 1A depicts the histological findings of the placenta of GDM women. Results revealed increasing syncytial knots and chorangiosis, indicating that the placenta may not be functioning properly in supporting the growing fetus. Besides, using immunohistochemistry staining, the expression of LOX-1 was increased 3.4-fold in GDM (Figure 1B,C).

### 3.2. Elevated Mitochondrial Fusion and Decreased Mitochondria Mass in GDM Placenta

**Figure 2:**
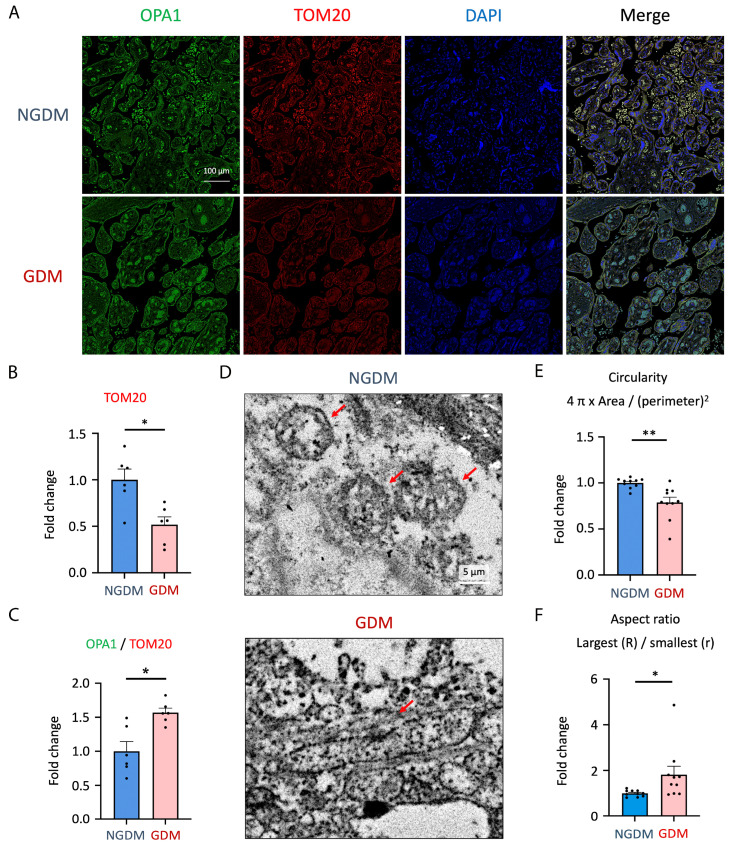
The trophoblast of the GDM placenta showed lower mitochondrial mass and enhanced mitochondrial fusion. (**A**) Confocal microscopy of the NGDM (**upper panel**) and GDM (**lower panel**) placenta. OPA1 controls mitochondrial fusion (green color), TOM20 depicts mitochondrial content (red color), and DAPI stains the background nuclei (blue color) (*n* = 6). Scale bar: 100 µm. (**B**) The fold change of TOM20 and (**C**) OPA1/TOM20 ratio was assessed according to the relative fluorescence signal intensity (*n* = 6). Data were quantified by ImageJ software version 1.54i. (**D**) Transmission electron microscopy (TEM) was used to examine mitochondrial structure and morphology in NGDM (**upper panel**) and GDM (**lower panel**) placenta. Scale bar in the representative Figure: 5 µm. The red arrow represents the location of mitochondria. (**E**) The fold change of circularity (4π × area/(perimeter))^2^ and (**F**) aspect ratio (largest (R)/smallest (r)) of mitochondria in NGDM and GDM placenta (*n* = 10 fields under transmission electron microscopy). * *p* < 0.05, ** *p* < 0.01. Abbreviations: GDM: gestational diabetes mellitus; NGDM: non-gestational diabetes healthy controls; OPA1: optic atrophy 1; TOM20: translocase of outer mitochondrial membrane 20; DAPI: 4′,6-diamidino-2-phenylindole; TEM: transmission electron microscopy.

LOX-1 is a scavenger receptor triggered by oxidized LDL (oxLDL) and electronegative LDL [38]. Activating the LOX-1 signaling pathway elevates oxidative stress and induces mitochondrial damage, leading to inflammation [39,40]. We thus further utilized confocal and transmission electron microscopy (TEM) to evaluate mitochondria expression levels and structure in the placenta of GDM. Results showed that mitochondria in the placenta of GDM have lower expression levels of TOM20 and higher OPA1/TOM20 ratios than the NGDM group, indicating lower mitochondrial mass and enhanced mitochondrial fusion. The mitochondrial mass decreased by 48.2%, and the OPA1/TOM20 ratio increased by 56.9% (Figure 2A–C).

Besides, TEM data indicated that the mitochondrial morphology was elongated in the placenta of GDM (Figure 2D). We calculated the circularity (4π × area/(perimeter))^2^ and aspect ratio (largest diameter (R)/smallest diameter (r)); results have also demonstrated that GDM placenta had enhanced mitochondrial fusion (Figure 2E,F).

### 3.3. Lysophosphatidylcholine Increased in GDM Cord Blood

**Figure 3:**
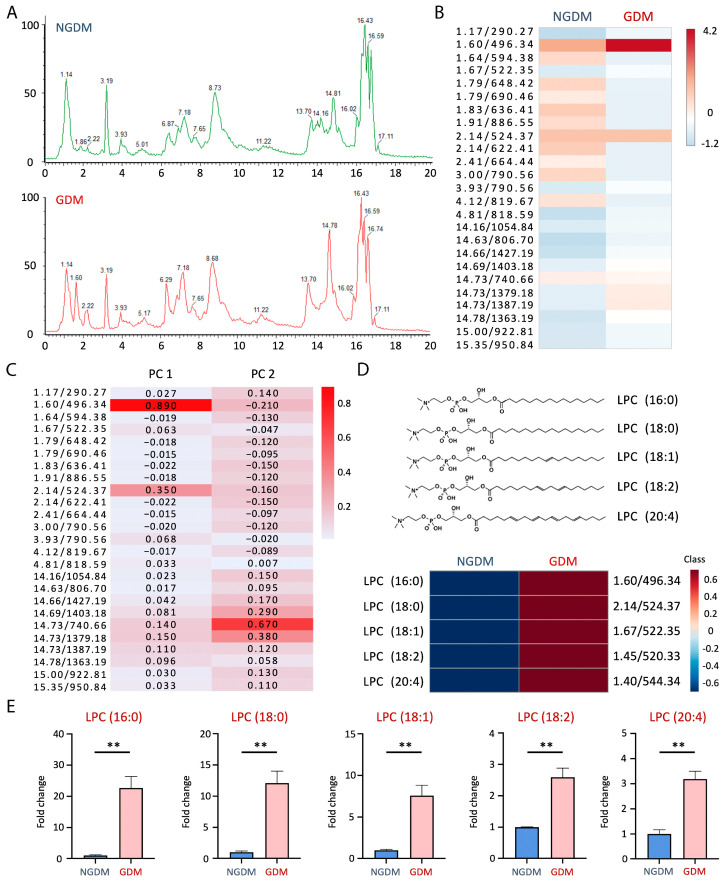
Lysophosphatidylcholine increased in GDM cord blood. (**A**) Representative lipid patterns in the cord blood of NGDM (**upper panel**) and GDM (**lower panel**) as determined by UPLC/MS^E^ (*n* = 6 for the NGDM group, *n* = 6 for the GDM group; lipid extracts were from the cord blood of 12 Taiwanese women). Total lipids were separated using a CSH C18 column (Waters Corporation; Milford, MA, USA). Furthermore, the mass-to-charge signals were detected in the positive mode using XEVO G2 QTOF mass spectrometry (Waters Corporation; Milford, MA, USA). (**B**) We chose *m*/*z* with (1) abundance > 5000, (2) *p* < 0.0001, (3) fold change > 5 as the criteria and selected the top 25 differentiative markers for GDM lipids. (**C**) By principal component analysis (PCA), PC1 and PC2 were determined. Furthermore, the most critical components in PC1 and PC2 were calculated, respectively. (**D**) According to the retention time and fragment daughter ions of lipid standards, we identified LPC signals, and (**E**) calculated the fold change of GDM in comparison to NGDM. ** *p* < 0.01. Abbreviations: GDM: gestational diabetes mellitus; NGDM: non-gestational diabetes healthy controls; LPC: lysophosphatidylcholine; UPLC: ultra-pure liquid chromatography; MS^E^: mass spectrometry in data-independent analysis mode; CSH: charged surface hybrid.

We have previously shown that electronegative LDL, a mild oxidized LDL isolated from humans, is elevated in the maternal and cord blood of GDM. In this work, we go further into the lipid composition to better understand the pathogenesis of GDM. Total lipids were separated using a CSH C18 column (Waters Corporation; Milford, MA, USA). Furthermore, the mass-to-charge signals were detected in the positive mode using XEVO G2 QTOF mass spectrometry (Waters Corporation; Milford, MA, USA) (Figure 3A). We identified the top 25 differentiative markers for GDM lipids based on *m*/*z* criteria of (1) abundance > 5000, (2) *p* < 0.0001, and (3) fold change > 5 (Figure 3B).

Principal component analysis (PCA) for lipid components was used to differentiate GDM and NGDM, mainly determined by PC1 and PC2. GDM cord blood contained significantly higher levels of PC1 components. The average score of PC1 was 120,000 ± 60,000 in GDM and −120,000 ± 2000 in NGDM. In contrast, the average score of PC2 was 2000 ± 300 in GDM and −1000 ± 200 in NGDM. In addition, the most relevant PC1 components were 1.60/496.34 and 2.14/524.38 (retention time/mass to charge ratio), with component importance values of 0.89 and 0.35, respectively (Figure 3C). The most significant components in PC2 were 14.73/740.66 and 14.73/1379.18 (retention time/mass-to-charge ratio), with component importance values of 0.67 and 0.38 (Figure 3C).

According to the facts above, the PC1 determined by mass spectrometry and PCA best distinguishes GDM and NGDM. Furthermore, the most prominent lipid molecules, 496.34 and 524.38 *m*/*z*, were significantly enhanced in GDM. We matched these signals based on the retention time and fragment daughter ions and then identified 496.34 *m*/*z* as LPC (16:0) and 524.38 *m*/*z* as LPC (18:0) (Figure 3D). By means of relative quantification, LPC (16:0) was 22.7 times higher in the cord blood of GDM than in NGDM, and LPC (18:0) was 12.1 times higher (Figure 3E).

### 3.4. LPC Induces Excessive Mitochondrial ROS Production

**Figure 4:**
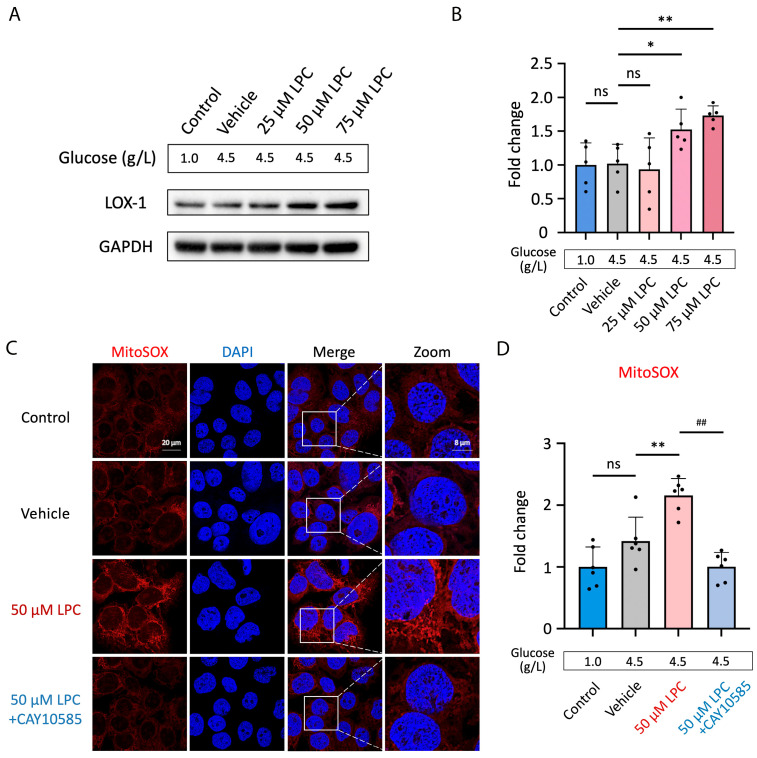
LPC-induced LOX-1 overexpression and excessive mitochondrial ROS production. (**A**) Human placental JEG3 cells were challenged with 25, 50, or 75 μM LPC. The glucose levels were 1.0 g/L for non-treat control and 4.5 g/L for vehicle control and other treatments. LOX-1 expression was evaluated using Western blot testing. (**B**) The fold changes of LOX-1 expression were calculated by chemiluminescence signal intensity (*n* = 5). Data were quantified using ImageJ software, and the differences were measured using one-way ANOVA. (**C**) 50 μM LPC was treated to human placental JEG3 cells with or without 2.5 μM CAY10585, the HIF-1α inhibitor, for 24 h. The mitochondria ROS production was detected by staining with MitoSOX^TM^ (red color). The nuclei were stained with DAPI (blue). Signals were visualized by fluorescence microscopy. Scale bar, 20 µm. (**D**) The fold changes of the fluorescence signal were measured according to fluorescence signal intensity (*n* = 6). Data were quantified by ImageJ software. * *p* < 0.05, ** *p* < 0.01, data were compared to control; ^##^ *p* < 0.01, data were compared to 50 μM LPC treatment. Abbreviations: LPC: Lysophosphatidylcholine; LOX-1: lectin-like oxidized low-density lipoprotein (LDL) receptor-1; HIF-1α: Hypoxia-inducible factor 1-alpha; ROS: reactive oxygen species; MitoSOX: mitochondrial superoxide indicators for live cell imaging; DAPI: 4′,6-diamidino-2-phenylindole, a fluorescent stain that binds strongly to adenine–thymine-rich regions in DNA; ANOVA: analysis of variance, ns: no significant differences.

In order to investigate the impact of LPC on LOX-1 expression, we challenged human placental JEG3 cells with 25, 50, or 75 μM LPC. Glucose levels were 1.0 g/L for the non-treatment group and 4.5 g/L for the vehicle control and other treatments. While the JEG3 cells treated with 50 μM or 75 μM LPC, the LOX-1 expression was significantly increased in a dose-dependent manner (50 μM LPC vs. vehicle control, *p* < 0.05; 75 μM LPC vs. vehicle control, *p* < 0.01, Figure 4A,B). Because the alterations in mitochondrial structure result from adaptive responses to oxidative stress, which are mediated by the transcription factor HIF-1α [41], we assessed mitochondrial oxidative stress in response to LPC treatment and examined its relationship to HIF-1α signaling. Results demonstrated that the JEG3 cells exposed to 50 μM LPC generated increasing reactive oxygen species (ROS) (*p* < 0.01, Figure 4C,D). Co-treatment with CAY10585, a HIF-1α inhibitor, significantly reduced ROS production compared to LPC treatment alone (*p* < 0.01).

### 3.5. LPC Impairs Mitochondrial Homeostasis through HIF-1α Pathway

**Figure 5:**
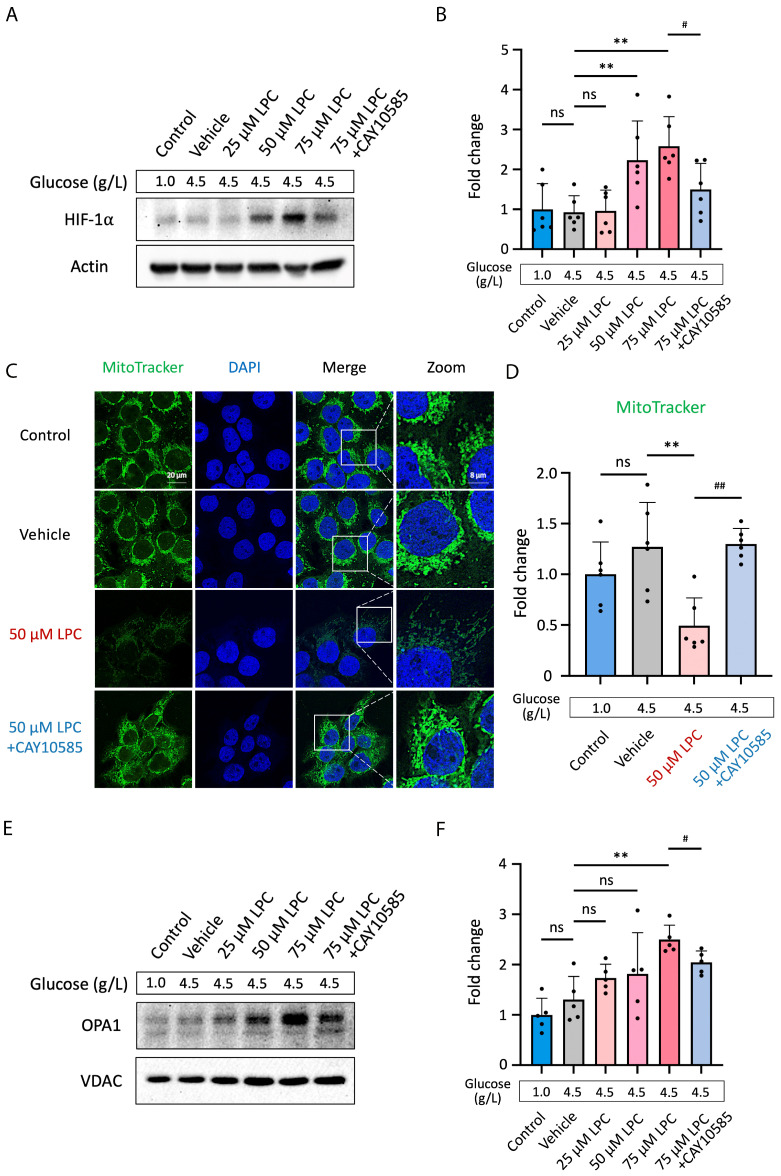
Lysophosphatidylcholine disrupts mitochondrial homeostasis via the HIF-1α pathway. (**A**) Human placental JEG3 cells were treated with 25, 50, or 75 μM LPC to assess its effect on HIF-1α signaling. Besides, JEG3 cells were cotreated with 75 μM LPC and 2.5 μM CAY10585, the HIF-1α inhibitor, for 24 h. The glucose levels were 1.0 g/L for non-treat control and 4.5 g/L for vehicle control and other treatments. (**B**) Data were quantified by ImageJ software. The fold changes of signal intensity were tested (*n* = 6). (**C**) The mitochondrial mass of JEG3 cells was determined using the signal intensity of MitoTracker^TM^ Green FM labeling. Scale bar, 20 µm. (**D**) Fluorescence signal intensity was measured (*n* = 6). (**E**) Finally, the expression of OPA1 was tested for mitochondrial fusion. (**F**) Data were quantified by ImageJ software (*n* = 5). ** *p* < 0.01, data were compared to vehicle control; ^##^ *p* < 0.01, data were compared to 75 or 50 μM LPC treatment (# for inhibitor effect). Abbreviations: LPC: Lysophosphatidylcholine; HIF-1α: Hypoxia-inducible factor 1-alpha; MitoTracker^TM^ Green FM: enable mitochondria visualization with fluorescent imaging; DAPI: 4′,6-diamidino-2-phenylindole, a fluorescent stain that binds strongly to adenine–thymine-rich regions in DNA; ns: no significant differences.

To further understand the detailed pathomechanism of LPC on placental cells, we evaluated its impact on HIF-1α and mitochondrial fission-fusion dynamics. Human placental JEG3 cells were exposed to 25, 50, or 75 μM LPC. Combining LPC with CAY10585 led to the inhibitory control. Results showed that the HIF-1α accumulation was shown to be dose-dependently triggered by 50 or 75 μM LPC (50 μM LPC vs. vehicle control, *p* < 0.05; 75 μM LPC vs. vehicle control, *p* < 0.01, Figure 5A,B). The HIF-1α expression was down-regulated (*p* < 0.01, Figure 5A,B) when co-treated with CAY10585.

MitoTracker^TM^ Green FM was utilized to label the mitochondrial matrix in JEG3 cells in order to examine the impact of LPC on mitochondrial mass [42]. LPC at 50 μM reduced mitochondria mass by 62.2% compared to the vehicle control (*p* < 0.01). While co-treated 50 μM LPC and CAY10585 to the JEG3 cells, the MitoTrackerTM signals were reversed. The signal intensity was 2.6 times higher in 50 μM LPC and CAY10585 treatment when compared to 50 μM LPC alone treatment (*p* < 0.01, Figure 5C,D). Observe that Figure 5C, right panel “ZOOM”, shows elongation of the mitochondria treated with 50 μM LPC.

OPA1 resides in the inner membrane of the mitochondria, which helps control the stability and energy production of the mitochondria. In addition, OPA1 promotes mitochondrial fusion and contributes to the mechanism of membrane remodeling [43]. Therefore, we assess OPA1 protein levels by western blot testing to determine if LPC treatments regulate OPA1 expression through HIF-1α signaling. Human placental JEG3 cells were challenged with 25, 50, or 75 μM LPC for 24 h. The non-treatment group’s glucose levels were 1.0 g/L, whereas the vehicle control and other treatments had levels of 4.5 g/L. Co-treatment of LPC and CAY10585 was the inhibitory control. The findings demonstrated that the OPA1 expression was significantly enhanced by 75 μM LPC (*p* < 0.01, Figure 5E,F). The HIF-1α inhibitor, CAY10585, down-regulates the LPC effects on OPA1 expression (*p* < 0.01, Figure 5E,F).

### 3.6. LPC Impaired Mitochondria Electron Transport Chain of GDM Trophoblast

**Figure 6:**
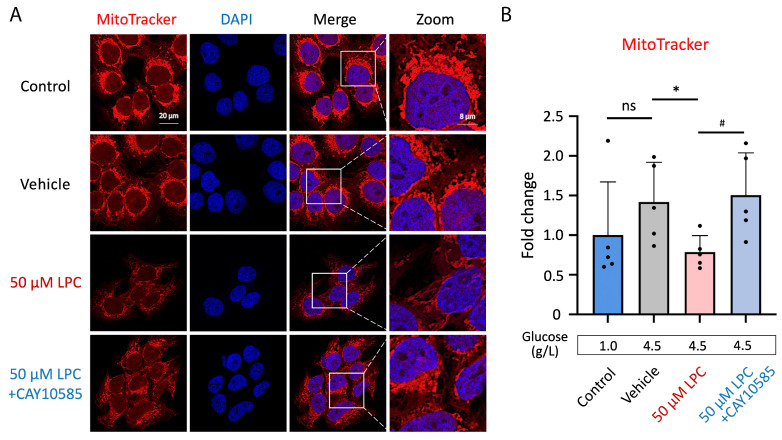
Lysophosphatidylcholine impaired the mitochondrial electron transport chain function. (**A**) Human placental JEG3 cells were treated with 50 μM LPC to evaluate the mitochondrial function. Besides, JEG3 cells were cotreated with 50 μM LPC and 2.5 μM CAY10585, the HIF-1α inhibitor, for 24 h (*n* = 6). The glucose levels were 1.0 g/L for non-treat control and 4.5 g/L for vehicle control and other treatments. Scale bar, 20 µm. (**B**) Fluorescence signal intensity was measured. Data were quantified by ImageJ software. The fold changes of signal intensity were tested (*n* = 5). * *p* < 0.05, data were compared to vehicle control; ^#^ *p* < 0.05, data were compared to 50 μM LPC treatment. Abbreviations: LPC: Lysophosphatidylcholine; HIF-1α: Hypoxia-inducible factor 1-alpha; MitoTracker^TM^ Red FM: fluorescent signal intensity was dependent on mitochondrial potential; DAPI: 4′,6-diamidino-2-phenylindole, a fluorescent stain that binds strongly to adenine–thymine-rich regions in DNA; ns: no significant differences.

In order to investigate the effect of LPC on mitochondrial function, the mitochondrial electron transport chain in JEG3 cells was labeled with MitoTracker^TM^ Red FM [44]. 50 μM LPC reduced fluorescence signals of the electron transport chain by 44.5%; data were compared to the vehicle control (*p* < 0.05). While co-treated 50 μM LPC and CAY10585, the MitoTracker^TM^ Red FM signals were reversed (*p* < 0.01, Figure 6A,B).

## 4. Discussion

In the current study, we found that the cord blood of GDM fetuses showed enhancing levels of LPC, including LPC (16:0), which was 22.7 times more than in non-GDM controls, and LPC (18:0), which was 12.1 times higher (Figure 3). LPC is the key factor positively correlated with cardiovascular diseases (CVD) and neurological disorders [45]. In circulation, LPC is mainly produced through the degradation of phosphatidylcholine by lipoprotein-associated phospholipase A_2_ (Lp-PLA_2_). A greater quantity of LPC causes plaque inflammation and vulnerability [46]. Simultaneously, overexpression of Lp-PLA_2_ contributes to medial arterial calcification and vascular remodeling [47]. The LPC effects are signaling through an orphan G protein receptor 2-mediated (G2A) signaling pathway, which results in increased production of intercellular adhesion molecule-1 (ICAM-1) and vascular cell adhesion molecule-1 (VCAM-1) for inflammatory responses such as T cell activation and leukocyte recruitment [48]. Besides, LPC is enriched in oxLDL and electronegative LDL, which promote vascular endothelial cell damage and platelet activation via LOX-1 signaling [20,49,50].

Previous studies have shown that the placenta exhibits the highest level of LOX-1 expression among various human organs [51]. Here, we further demonstrated that the LOX-1 expression could be enhanced in the placenta of GDM patients (Figure 1B,C), which was positively correlated with a higher percentage of syncytial knots and chorangiosis (Figure 1A). LOX-1 was initially cloned and recognized as the receptor of oxLDL that is expressed on vascular endothelial cells [52]. Through LOX-1, oxLDL downregulates fibroblast-growth factor-2 (FGF-2) and inhibits the phosphatidylinositol 3-kinase (PI3K)/protein kinase B (AKT) pathway, leading to mitochondria impairment and endothelial cell apoptosis [53]. Later, LOX-1 has been reported on various cells such as macrophages, platelets, smooth muscle cells, adipocytes, hepatocytes, and cancer cells [54,55,56,57]. The pathophysiology of atherosclerosis is caused by the interaction of C-reactive protein (CRP), atherogenic LDL, and LOX-1 [19]. In the placenta of pregnant women, overexpressed LOX-1 is associated with fetoplacental vascular dysfunction in pre-eclampsia. LOX-1 enhances ROS and reduces intracellular nitric oxide (NO), leading to apoptosis of syncytiotrophoblast cells [58].

The treatment of LPC in human placental JEG3 cells increased LOX-1 expression (Figure 4A,B). The findings are consistent with previous investigations [59,60], suggesting that the GDM placenta may have a high affinity to the LPC, oxLDL, and electronegative LDL, uptaking more harmful lipids. In addition, LOX-1 overexpression was involved in the pathology of GDM and associated with pregnancy complications like pre-eclampsia pathogenesis [60,61].

LPC deposit in placental tissue contributes to increased mitochondrial ROS formation and HIF-1α upregulation (Figure 4 and Figure 5A,B). Because of increased oxidative stress, LPC-treated JEG-3 cells displayed prolonged mitochondria morphology and disrupted mitochondrial homeostasis (Figure 5E,F), leading to mitochondrial dysfunction (Figure 6A,B). These results are consistent with findings of clinical GDM placental specimens (Figure 2D–F)**.** In addition to possessing higher LPC concentration in the cord blood, cytosolic phospholipase A_2_ (cPLA_2_) and Ca^2+^-independent phospholipase A_2_ (iPLA_2_) also contribute to the breakdown of phosphatidylcholine, allowing LPC to be overproduced intracellularly [62,63]. Previous studies have shown that cPLA2 is overexpressed in the vessels of advanced-stage cardiovascular disease patients; it has emerged as a novel therapeutic target to prevent vascular calcification in CVD [64]. In addition, iPLA2 is involved in the translocation of HIF-1α to the nucleus for hypoxia or inflammatory responses [65].

The study design aimed to mimic the GDM placenta and determine whether high glucose or LPC contributes the best to the harmful effects on mitochondria. In our study, the glucose level for the non-treatment group was 1.0 g/L. On the other hand, it was 4.5 g/L for the vehicle control, LPC treatments, and co-treatment of 75 μM LPC with 2.5 μM CAY10585. Results showed that the non-treatment group was no different from vehicle control, whereas LPC dose-dependently enhances the LOX-1 and OPA-1 expression (Figure 4A,B and Figure 5E,F) and HIF-1α upregulation (Figure 5A,B). Hyperglycemia is a highly concerning medical issue because it can result in trophoblast dysregulation and aberrant placentation. Additionally, high glucose enhances vascular endothelial cell senescence and malfunction [66,67], contributing to an upward medical cost trajectory. The current GDM treatment mainly focuses on glucose management, such as medical nutrition therapy and insulin therapy [68]. Here, we have demonstrated that LPC is another contributor to GDM complications. Novel therapies for diabetes mellitus in pregnancy are required, with an emphasis on lipid metabolism and LPC clearance in particular.

**Figure 7:**
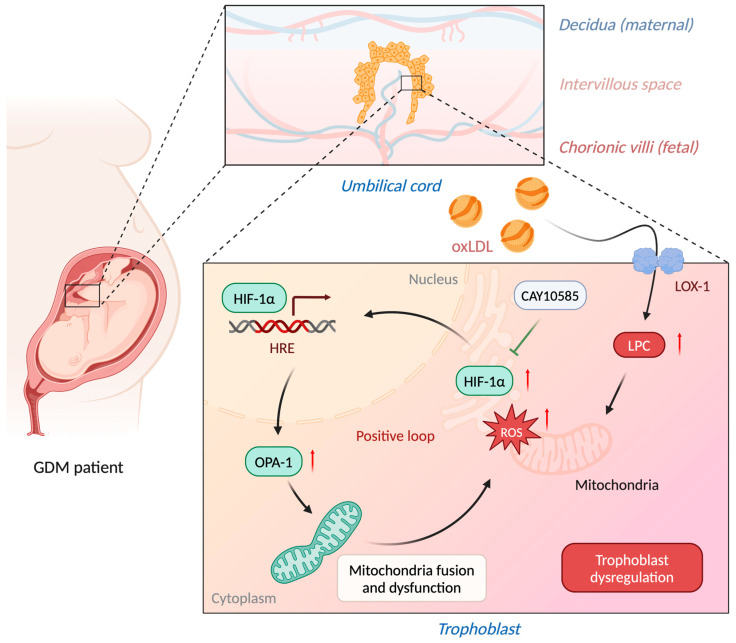
A schematic diagram showing the mechanism by which LPC triggers oxidative stress, mitochondria fusion, and dysfunction, which in turn causes trophoblast dysregulation and is positively connected with a greater incidence of chorangiosis and syncytial knots in GDM. The red arrow indicates the increasing levels of LPC, ROS, HIF-1α, and OPA-1. Figures created with BioRender.com. Abbreviations: GDM: gestational diabetes mellitus; LPC: lysophosphatidylcholine; oxLDL: oxidized low-density lipoprotein; LOX-1: lectin-like oxidized low-density lipoprotein (LDL) receptor-1; HIF-1α: hypoxia-inducible factor 1-alpha; ROS: reactive oxygen species; OPA1: optic atrophy 1.

To sum up, LPC plays an important role in the pathogenesis of GDM (Figure 7). The placenta is the primary organ that mediates maternal–fetal exchange [69]. Despite the fetus being available for de novo lipogenesis by the fetal liver, the lipid source still greatly depends on maternal-fetal communications [70]. LPC is one of the major lipid components in oxLDL and electronegative LDL [71]. Through LOX-1, LPC may accumulate in the placenta of GDM cases. LPC disrupts the trophoblast mitochondria function through ROS overproduction, HIF-1α signaling, and OPA-1 overexpression. It forms a positive loop that worsens the placental function and correlates to adverse pregnancy outcomes.

## 5. Conclusions

In conclusion, highly elevated LPC, such as LPC (16:0) and LPC (18:0) in cord blood, plays a pivotal role in GDM. It enhances the mitochondrial ROS and upregulates the HIF-1α expression, contributing to trophoblast impairment and pregnancy complications.

## Figures and Tables

**Table 1:** Demographic data of 12 Taiwanese women enrolled in this study

	NGDM (*n* = 6)	GDM (*n* = 6)	*p*-Value
Age, y (mean ± SD)	34.5 ± 2.88	33.5 ± 5.13	0.7316
Fasting glucose	83.3 ± 3.5	96.3 ± 6.7	** 0.0022
1-h OGTT	124.0 ± 16.1	160.0 ± 39.4	0.1797
2-h OGTT	120.0 ± 15.5	141.0 ± 32.4	0.4567

Abbreviation: NGDM: non-gestational diabetes mellitus; GDM: gestational diabetes mellitus; SD: standard deviation; 1-h OGTT: one-hour 75 g oral glucose tolerance test; 2-h OGTT: two-hour 75 g oral glucose tolerance test. ** *p* < 0.01.

## Data Availability

Data is contained within the article.

## Abbreviations

ACN	acetonitrile
AKT	protein kinase B
ApoE	apolipoprotein E
*apoE* ^−/−^	apolipoprotein E-deficient
ASCVD	atherosclerotic cardiovascular disease
BCRC	Bioresource Collection and Research Center
cPLA_2_	cytosolic phospholipase A_2_
CVD	cardiovascular disease
CRP	C-reactive protein
CSH	charged surface hybrid
DAB	3,3′-diaminobenzidine
DAPI	4′,6-diamidino-2-phenylindole
DG	diacylglycerol
EDTA	ethylenediaminetetraacetic acid
ER	endoplasmic reticulum
ESI	electrospray ionization interface
EVT	extravillous trophoblast
FGF-2	fibroblast-growth factor-2
GDM	gestational diabetes mellitus
H&E	Hematoxylin and eosin
HDL-C	high-density lipoprotein cholesterol
HIF-1α	hypoxia-inducible factor-1α
HRP	horseradish peroxidase
IRB	institutional review board
IADPSG	International Association of Diabetes and Pregnancy Study Groups
ICAM-1	intercellular adhesion molecule-1
IgG	immunoglobulin *G* (IgG)
IPA	isopropanol
iPLA_2_	Ca^2+^-independent phospholipase A_2_
JEG3	choriocarcinoma cells
KMUH	Kaohsiung Medical University Hospital
L5-LDL	the most electronegative subfraction of low-density lipoprotein
LDL(−)	electronegative low-density lipoprotein
LDL-C	low-density lipoprotein-cholesterol
LDLR	low-density lipoprotein receptor
LC/MS	liquid chromatography/mass spectrometry
LC/MS^E^	liquid chromatography/data-independent collection mode mass spectrometry
LOX-1	lectin-like oxidized low-density lipoprotein receptor 1
LPA	lysophosphatidic acid
LPC	lysophosphatidylcholine
Lp-PLA2	lipoprotein-associated phospholipase A2
MEM	minimum essential media
MFSD2A	major facilitator superfamily domain-containing protein 2
MS	mass spectrometry
MS^E^	data-independent collection mode mass spectrometry
NGDM	non-gestational diabetes
NO	nitric oxide
NP-40	4-Nonylphenyl-polyethylene glycol, Nonidet P™-40
OGTT	oral glucose tolerance test
OPA1	mechanochemical GTPase optic atrophy 1
OsO4	osmium tetroxide
oxLDL	oxidized low-density lipoprotein
PAS	placenta accreta spectrum
PBS	phosphate-buffered saline
PI3K	phosphatidylinositol 3-kinase
PVDF	polyvinylidene difluoride membrane
RIPA	radioimmunoprecipitation assay buffer
ROS	reactive oxygen species
QTOF	quadrupole time of flight mass spectrometry
SDS	sodium dodecyl sulfate
SEM	standard error of the mean
SM	Sphingomyelin
T2D	type 2 diabetes mellitus
TC	total cholesterol
TEM	transmission electron microscopy (TEM)
TEMED	N, N, N′, N′-Tetramethyl ethylenediamine
TG	triglyceride
TOM20	mitochondrial import receptor subunit
Tris-HCl	TRIS Hydrochloride
UPLC	ultra-performance liquid chromatography
VCAM	vascular cell adhesion molecule-1
VDAC	voltage-dependent anion channel
